# Development of Strategies to Decrease False Positive Results in Newborn Screening

**DOI:** 10.3390/ijns6040084

**Published:** 2020-11-02

**Authors:** Sabrina Malvagia, Giulia Forni, Daniela Ombrone, Giancarlo la Marca

**Affiliations:** 1 Newborn Screening, Clinical Chemistry and Pharmacology Laboratory, Meyer Children’s University Hospital, 50139 Florence, Italy; sabrina.malvagia@meyer.it (S.M.); giualia.forni@meyer.it (G.F.); daniela.ombrone@meyer.it (D.O.); 2 Department of Experimental and Clinical Biomedical Sciences, University of Florence, 50139 Florence, Italy

**Keywords:** newborn screening, second-tier test, false positives, tandem mass spectrometry, inborn error of metabolism

## Abstract

The expansion of national newborn screening (NBS) programmes has provided significant benefits in the diagnosis and early treatment of several rare, heritable conditions, preventing adverse health outcomes for most affected infants. New technological developments have enabled the implementation of testing panel covering over 50 disorders. Consequently, the increment of false positive rate has led to a high number of healthy infants recalled for expensive and often invasive additional testing, opening a debate about the harm-benefit ratio of the expanded newborn screening. The false-positive rate represents a challenge for healthcare providers working in NBS systems. Here, we give an overview on the most commonly used strategies for decreasing the adverse effects due to inconclusive screening results. The focus is on NBS performance improvement through the implementation of analytical methods, the application of new and more informative biomarkers, and by using post-analytical interpretive tools. These strategies, used as part of the NBS process, can to enhance the positive predictive value of the test and reduce the parental anxiety and healthcare costs related to the unnecessary tests and procedures.

## 1. Introduction

There are significant long-term cost benefit advantages in diagnosing inherited metabolic disorders shortly after birth. Many conditions which if untreated are characterized by episodes of metabolic decompensation can be effectively managed through diet when diagnosis is made before the development of symptoms. An early diagnosis often prevents clinical complications that may be irreversible [1,2].

Newborn screening (NBS) plays a crucial role in preventive medicine. Advances in technology and more sensitive diagnostic tests allow infants with manageable congenital diseases to be identified and treated within days of birth [3,4].

With the advent of Tandem Mass Spectrometry (MS/MS), one of the most important advances in the field of NBS, a single dried blood spot (DBS) can be used to screen a wide range of conditions [5]. Simultaneous quantification of amino acids and acylcarnitines can now detect neonates at risk for more than forty metabolic disorders [6].

Moreover, conditions included in national NBS programs continue to increase, also thanks to the development of novel immunoassay and real-time PCR testing platforms [4,7,8].

Despite the high sensitivity and specificity of current methods employed in NBS, a positive screening result does not establish a definitive diagnosis and follow-up analytical tests are required. Positive newborns must be recalled for clinical evaluation and additional laboratory tests. Given that NBS programs now include many more disorders requiring more tests than in the past, the rate of false positive results has increased [9].

The sensitivity and specificity of NBS tests are dependent on decisions regarding cut-off values. A false negative is usually more harmful than a false positive since an affected newborn may be missed. Adjusting cut off values to reduce the risk of false negatives often has the effect of increasing the false positive rate. Screening may, therefore, lead to a high number of unnecessary recalls and additional testing which can cause a non-optimal allocation of healthcare resources and parental anxiety with long-term consequences on the child–parent relationship [10]. 

An evaluation of parental stress highlighted significantly higher levels of anxiety in mothers with newborns in the false-positive group than in mothers whose infants tested negative. The mothers’ anxiety did not fully resolve after further testing eliminated the possibility of illness. Infants in the false positive group were more likely to be taken to emergency rooms and to be hospitalized than those who were not recalled at birth [11,12].

Given the psychological and economic impact of false positives, significant efforts have been made to improve the performance of NBS assays.

## 2. Introduction of New Disease-Specific Biomarkers: The Case of Tyrosinemia Type I

The most effective diagnostic markers are specific metabolites produced by a disease which are not present in healthy people.

The production of succinylacetone (SUAC) is caused by a deficiency of the enzyme fumarylacetoacetate hydrolase in the tyrosine catabolism pathway. The presence of SUAC in blood or urine is pathognomonic for Hereditary Tyrosinemia type I (HT-1). 

In the past, SUAC could not be extracted from DBS samples prepared according to classical protocols. Thus, the amino acid tyrosine was used as the metabolic marker for HT-1, which resulted in a high false positive rate due to the high incidence of benign transient Tyrosinemia in the neonatal population. Moreover, newborns affected by HT-1 can have normal tyrosine levels at birth, which leads to a high risk of false negative results and misdiagnoses [13,14,15]. 

To resolve this problem, hydrazine can be added to the alcoholic extraction solution, allowing the formation of hydrazone from hydrazine and a carbonyl compound of SUAC, which can be extracted from the sample matrix at the same time as amino acids and acylcarnitines [16]. Most laboratories have now introduced testing for the presence of SUAC in routine newborn screening protocols, which has reduced false negatives for HT-1 [17]. By selecting appropriate tyrosine cut off levels, other forms of Tyrosinemia (Type II and III) can be identified in which very high levels of tyrosine are present in blood soon after birth. This approach significantly reduces the false positive rate, optimizing resources and avoiding adverse psychological effects, including family stress. The adoption of the SUAC biomarker in the screening panel is strongly recommended [18,19]. 

## 3. Second-Tier Test Strategy 

**Figure 1:**
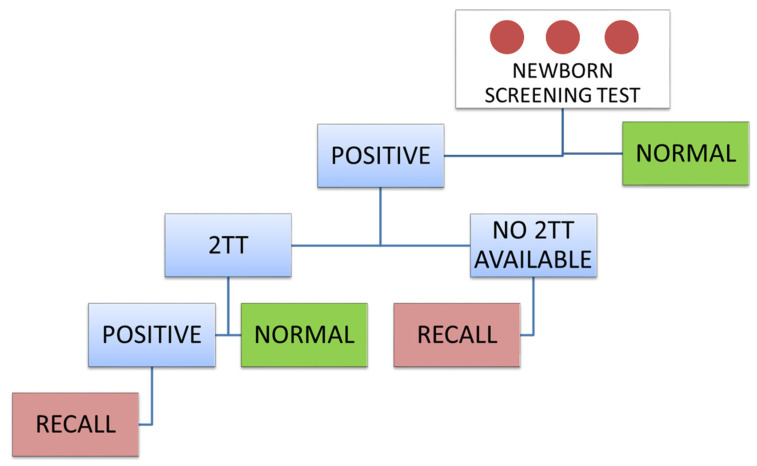
Flow-chart for newborn screening analytical process. When a 2-TT is available, the specificity of newborn screening is significantly improved. It may also be possible to reduce primary test cut-off to improve sensitivity.

An initial out of range screening result can be due to transient conditions, genetic variants, carrier status or overlap between the healthy and diseased population. Development of effective second-tier tests (2-TT) can minimize false positives and avoid unnecessary recalls [20]. A 2-TT is carried out when a primary screening result is abnormal (Figure 1). Positive specimens can be reanalysed by a more specific test based on different methodology. The 2-TT result confirms or overrules the primary screening result. These relatively expensive and time-consuming methods are unsuitable as first-tier screening tests for which a large number of samples must be processed. However, they are very effective for selected high-risk samples, improving the positive predictive value (PPV) of NBS programs.

A 2-TT can identify the same target as primary screening but with improved specificity thanks to separation from isomers or interfering substances, or it can screen for another diagnostic marker not included in first tier-screening. 

Several authors have estimated the extent to which 2-TT based strategies can reduce economic costs associated with false-positives. Gavrilov and colleagues report that in the absence of a 2-TT program, the total average cost of the first instance of follow-up for an abnormal C3-carnitine or elevated methionine is approximately $1500 per false positive [21]. The National Newborn Screening and Global Resource Center calculated that introducing a 2-TT by LC-MS/MS for adrenal steroids following a positive immunoassay-based 17-OHP screening result reduced costs by 85% [22].

### 3.1. Maple Syrup Urine Disease (MSUD)

Compared to chromatography-MS/MS, flow injection-MS/MS analysis (FIA-MS/MS) for expanded NBS has important advantages. It is fast, cheap and allows for high-throughput screening. However, it is unable to differentiate isobaric isoforms. The application of a 2-TT, using chromatographic separation prior to mass spectrometric detection, can be a useful in confirming a positive first-tier result, before the newborn is recalled.

A perfect example of how isobaric interferences may affect screening performance is in the combined detection of several branched-chain amino acids (BCAAs) in maple syrup urine disease (MSUD). Babies with MSUD accumulate leucine, isoleucine, valine and allo-isoleucine in blood and tissues with devastating effects [23]. Early diagnosis is essential for introducing dietary treatment which is associated with better long-term outcomes and lower mortality rates.

**Figure 2:**
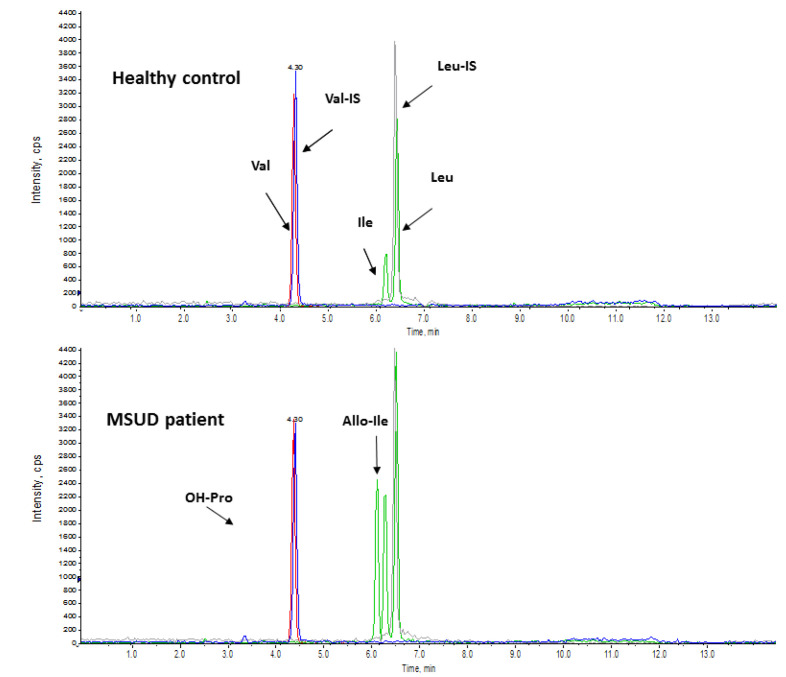
Chromatographic separation of branched-chain amino acids on DBS from a healthy control (**A**) and from a MSUD patient (**B**). The application of LC-MS/MS based testing allows several isobaric compounds, including Alloisoleucine (Allo-Iso), the pathognomic biomarker of MSUD to be identified.

The routine screening method measures several isobaric species of different amino acids (isoleucine, leucine, allo-isoleucine and OH-proline) combined in one analytical signal named “X-Leu”. However, this combined signal can be elevated in newborns receiving parenteral nutrition containing BCAA or in the clinically benign condition of hydroxyprolinemia and so 2-TT is necessary to quantify allo-isoleucine, a pathognomonic marker of MSUD disease. In healthy newborns allo-isoleucine is undetectable so affected babies can be clearly distinguished, meaning that unwarranted follow-up tests to rule out MSUD are unnecessary (Figure 2). 

Unfortunately, NBS can fail to detect the intermediate form of MSUD, which often only manifests in biochemical alterations during metabolic decompensation. Although the introduction of 2-TT can increase sensitivity by lowering the X-Leu cut off, some affected children cannot be identified at birth [24,25]. 

### 3.2. Isovaleric Acidaemia

An increase in C5-acylcarnitine (C5) levels in an NBS acylcarnitine profile can suggest both isovaleric acidaemia (IVA) with elevated isovalerylcarnitine or short/branched chain acyl-CoA dehydrogenase deficiency (SBCADD) with elevated 2-methylbutyrylcarnitine (2MBC). While the latter is an asymptomatic condition not included in worldwide NBS panels, IVA is a serious inherited condition caused by a defect in the catabolic pathway of leucine.

Several papers report on an increased recall rate in newborns due to elevated C5 caused by the intake of pivalic acid derivatives. Pivalic acid contained in some antibiotics or nipple creams can conjugate to free carnitine and became a C5-carnitine isobaric compound [26,27,28]. In these babies, organic acid analysis in urine is normal and subsequent analysis on DBS always shows a spontaneous decrease in C5 after the suspension of antibiotic treatment or nipple cream application.

**Figure 3:**
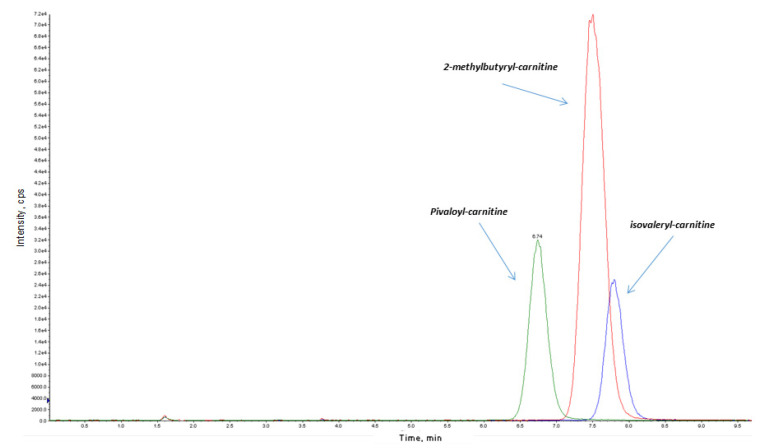
The 2-TT to reduce the false positive rate in expanded newborn screening for isovaleric acidemia (IVA). The contributions from different isobaric molecules can be separated by chromatography coupled with MS-MS as described in Poggiali et al. [26]. Interference causing false abnormal C5-acylcarnitine levels can be observed for pivalic acid supplementation or for 2-methylbutyrylglycinuria, a benign metabolic condition.

A chromatographic separation in NBS samples with elevated C5 allows for the identification of different isomers underlying abnormality before alerting the family (Figure 3). With 2-TT, PPV is near 100% based on the C5 cut off adjustment. However, as reported in the literature [27,28], since true cases are limited, more precise statistics will only be available when more data are collected.

### 3.3. Propionate Metabolism 

Not all the analytes included in expanded NBS have a pathognomonic significance and some can produce a high number of false-positive results [29].

High levels of propionylcarnitine (C3) in newborn screening may suggest an inborn error of propionate metabolism including propionic aciduria (PA) and methylmalonic aciduria (MMA) [30].

Children affected by PA and MMA are at risk of acute metabolic decompensation and detected newborns must be immediately recalled. Samples with elevated C3 can lead to a high false positive rate because concentrations found in affected newborns overlap with healthy individuals, reducing the marker’s specificity. However, if the C3 threshold is set too high, many affected babies can be missed [31].

The application of a 2-TT able to identify free organic acids such as methylmalonic acid (MMA) and propionic acid (3OH-PA) on DBS has greatly improved the PPV of C3 [32]. Sample preparation is rapid, without any derivatization step, and after a fast chromatography, free 3OH-PA and MMA are clearly separated from each other and differentiated from the isomeric forms, lactic acid and succinic acid [32,33].

It is not unusual for normal healthy infants to show small amounts of MMA in blood in the first days of life owing to hepatic enzyme immaturity; thus, inaccuracies in determining cut-off levels for MMA on DBS could lead, once again, to more false positives than expected. 

The new biomarker, heptadecanoylcarnitine C17 (C16:1-OH isobar), can be used to determine when 2-TT should be performed, even when the primary C3 marker falls within normal limits. It has been demonstrated that C17 is more sensitive than other C3 ratios in improving NBS diagnostic prediction rates [34].

### 3.4. Homocystinurias and Methylation Disorders

NBS laboratories use methionine as the primary marker for detecting homocystinuria and methylation disorders. Hypermethioninemia can be associated with classical homocystinuria, presenting elevated homocysteine in both blood and urine or with MAT I/III deficiency without homocysteinemia. Although evaluating homocysteine levels by a 2-TT on positive screening samples can be useful for patient management, there is no reduction in false positive rates, since an elevated methionine from an NBS always leads to patient recall. 

Low methionine levels can indicate an underlying re-methylation disorder, including severe MTHFR deficiency, typically associated with high homocysteine levels. Since methionine levels can be affected more than other amino acids, by poor sample storage, application of 2-TT for homocysteine quantification, when methionine on the first test is low, remains the most effective means of reducing false positives [35]. Many screening labs also use the ratio of methionine to phenylalanine as an additional parameter to reduce the need for recalls.

### 3.5. Congenital Adrenal Hyperplasia (CAH)

NBS for CAH, using the biomarker, 17-OH-progesterone (17-OHP), usually measured by fluoroimmunoassay on DBS, has been introduced in many states around the world. The levels of this hormone, which can be altered by the degree of prematurity and sampling time, are very variable in the first days of life, with the consequence that there is a high false positive rate [36].

Adjustments to cut-off values based on different gestational ages and/or on birth weights can help reduce the number of false positives. Moreover, as 17-OHP decreases over time in healthy newborns but increases in affected infants, some states have added a second screening test for all newborns at 8–14 days of age [37]. Fingerhut and colleagues reported a reduction of 40% in false positives with the introduction a 2-TT which involves the extraction of 17 OHP from positive first-screen DBS with diethyl ether in order to eliminate contamination from polar steroids (steroid sulfates) [38]. Recently, many laboratories have decided to perform a 2-TT using steroid hormone profiling by LC-MS/MS which can reveal the characteristic diagnostic pattern of elevated 17-OHP, high androstenedione level and low cortisol, thus improving the PPV of CAH screening programs [39,40].

### 3.6. Adenosine Deaminase Deficiency (Ada-SCID) and Purine Nucleoside Phosphorylase Deficiency (PNP-SCID)

Severe combined immunodeficiencies (SCIDs) are rare disorders, characterized by a congenital defect of the immune system, associated with poor prognosis if there is delay in diagnosis and treatment. In recent years, following the development of rapid and more accurate diagnostic methods together with more effective treatments, screening for SCIDs has been incorporated into many NBS programs. So far, the most sensitive and specific method for SCIDs screening is a quantitative polymerase Chain Reaction (PCR) to dose the copy number of T-cell receptor assay gene excision circles (TRECs) [41]. This method, however, is too expensive to be adopted in all countries. Moreover, in very premature babies and in patients with infection, TRECs can be significantly lower than in healthy subjects, generating a large number of false positives [42]. 

Two SCIDs, adenosine deaminase deficiency (ADA-SCID) and purine nucleoside phosphorylase deficiency (PNP-SCID), are characterized by enzyme defects in the purine salvage pathway, resulting in the accumulation of metabolites that are toxic to lymphoid lineage cells. Consequently, these two immunodeficiencies are also metabolic disorders and the increased production of purine metabolites can be identified by mass spectrometry on DBS. This method has the advantage of being cheap—the biomarkers can be included in the analyte panel during routine expanded newborn screening—and can thus be adopted where there are not sufficient funds for genetic screening by TREC testing.

It has been demonstrated that deoxyadenosine and deoxyguanosine (pathognomonic markers of ADA-SCID and PNP-SCID, respectively) are absent in healthy subjects. The test has a very high sensitivity and false positive results can be avoided by a prompt second-tier LC-MSMS method, able to separate any interferers [43,44].

## 4. Lysosomal Storage Disorders (LSD)

In the last fifteen years, screening for several LSDs has been included in several NBS programs. Analysis is performed by measuring lysosomal enzymatic activities on DBS using either MS/MS or fluorimetric assay [45]. Recently some countries have employed digital microfluidics platforms (DMF-F) for the NBS of Pompe, Fabry, Gaucher and MPSI [46,47]. 

Some ethical issues about expanding NBS for LSDs remain unresolved. One of the main problems is a lack of genotype-phenotype correlation. Many genetic alterations detected in lysosomal NBS programs have been associated with late-onset forms or have been identified as genetic variants of unknown significance (VUS). Moreover, the high frequency of pseudodeficiency alleles increases the rate of false positive results. 

To reduce unnecessary recalls some NBS laboratories carry out genotyping using residual DBS specimens for 2-TT analysis [48]. However, due to the unknown significance of many discovered mutations and the poor correlation between the level of residual enzyme activity and genotype, predicting clinical phenotypes remains a challenge and clinical protocols almost always require patients to be followed until the eventual appearance of symptoms. Despite these problems, however, the introduction of post-analytical tools and 2-TT tests for LSDs to increase the specificity of NBS appears to yield promising results [49].

Nevertheless, the efficacy of using biochemical biomarkers for LSD has yet to be clarified; data is lacking on biomarker concentrations in newborns with attenuated phenotypes and further studies must be conducted.

### 4.1. Pompe Disease

Worldwide NBS programs for Pompe disease (PD) are based on performing assays for enzymatic activity of acid α-glucosidase (GAA), one of the GAA isoforms.

The main cause of false positive results in Pompe NBS is the high prevalence of pseudodeficiency alleles in the newborn population. Pseudodeficiency variants decrease enzyme affinity on the artificial substrate used in testing, but have no effect on the natural substrate. In Taiwan, where the Pompe NBS program employs fluorescence assay, screening results have shown a high frequency of the pseudodeficiency allele p.G576S (14.5%), making the false positive rate unacceptable. To improve NBS performance, the ratio of neutral and acid α-glucosidase activities has been proposed as a cutoff to distinguish between true-positives and false-positives. Unfortunately, however, cut off values cannot distinguish between newborns with the milder form of PD and pseudodeficit carriers [50].

Recently, Tortorelli and colleagues have described a new biomarker employed in NBS for PD based on the measurement of the creatine/creatinine (Cre/Crn)/GAA ratio on DBS [51]. Quantification with isotope labelled internal standards of both Cre and Crn were conducted during expanded NBS without modifying the routinely used method [52]. The Cre/Crn ratio may be considered an early marker of muscle involvement and when integrated with GAA activity is a useful second-tier test to aid the interpretation of first-tier results [51].

### 4.2. Fabry Disease

Genetic testing on positive NBS samples for Fabry disease (FD) has revealed a high prevalence of neutral variants in the GLA gene. Whereas the diagnosis of male patients with the classical phenotype is usually straightforward, the clinical implications of GLA variants of uncertain significance remain incompletely understood [53].

In 2008, the deacetylated form of globotriaosylsphingosine (LysoGb3) was reported as a prognostic biomarker for FD [54]. All male patients with classical FD show high levels of LysoGb3 in plasma and it also appears to be increased in symptomatic female patients, although to a lesser extent than in male patients [55,56,57]. However, a significant percentage of FD affected females have GLA activity within the normal range rendering identification through NBS unreliable [58].

In the light of these findings sensitive analytical methods by LC-MS/MS have been developed to measure Lyso-Gb3 on DBS from FD patients [59]. Males with classical FD showed elevated Lyso-Gb3 levels. Males with late-onset GLA mutations and pre-symptomatic females were not statistically different from healthy controls. There was no apparent statistical difference in biomarker concentration between males with late-onset GLA mutations, pre-symptomatic females and healthy controls, highlighting how the use of LysoGb3 in NBS is very limited [49,59].

### 4.3. Mucopolysaccharidosis Type I (MPS I)

The deficient activity of α-iduronidase (IDUA), one of the lysosomal enzymes involved in the breakdown of glycosaminoglycans (GAGs), leads to the accumulation of heparan sulphate (HS) and dermatan sulphate (DS) in affected MPSI patients also before birth [60]. The early diagnosis of pre-symptomatic newborns is crucial to ensuring optimal outcomes following the prompt initiation of treatment [61].

In many NBS laboratories, second-tier genotyping analysis is carried out for newborns with low IDUA activity. However, because of the high frequency of private mutations and VUS, molecular analysis may fail to predict the clinical phenotype of pre-symptomatic newborns.

The application of LC-MS/MS technology to quantify DS and HS has allowed the development of a 2-TT on DBS samples to confirm or overrule first tier results and improve the diagnostic process [62,63,64]. GAGs analysis on DBS using LC-MS/MS has been shown to be a more effective second-tier test than molecular analysis and is becoming the most widely adopted 2-TT strategy in NBS for MPSI [65,66,67]. Promising data have been published on the identification of the accumulation of HS and DS on neonatal DBS from newborns affected by MPSI-H (severe form) and MPSI-H/S (intermediate form) [68]. However further studies are needed to evaluate the reliability and consistency of this method, especially for identifying late onset forms (MPSI-S).

### 4.4. Gaucher Disease

The NBS program for Gaucher disease (GD) is based on the measurement of lysosomal β- glucocerebrosidase (GBA) activity using tandem mass spectrometry or fluorimetric assay. In affected patients, GBA deficiency causes the accumulation of glucosylceramide in lysosomes of macrophages, called Gaucher cells.

Chitotriosidase and CCL18 are well-known Gaucher cell markers but they are non-specific and can also be elevated in other lysosomal storage disorders such as Niemann-Pick C1. Researchers have focused on discovering new biomarkers for the disease. High levels of glucosylsphingosine (lysoGb1) have been found in plasma samples of symptomatic GD patients [69]. Further studies have demonstrated the high sensitivity and specificity of lysoGb1 in distinguishing GD patients from healthy controls [70,71]. LysoGb1 concentrations correlate with genotype and disease burden: patients with severe phenotypes have significantly higher lysoGb1 concentrations compared to mildly affected patients [69,70]. Recently, this biomarker has been investigated in order to develop a 2-TT able to correctly predict diagnostic outcome in disease-suspected newborns. Currently, there are few available data and the prognostic power of this biomarker in pre-symptomatic patients remains uncertain [49].

### 4.5. Krabbe Disease

In 2006 newborn screening for Krabbe disease (KD) began in New York State to identify pre-symptomatic cases of the infantile disease and ensure prompt treatment. As previously described, the first-tier test is based on measuring the enzymatic activity of galactocerebrosidase (GALC) which plays an essential role in myelin turnover [72].

Genetic testing conducted on newborns with low GALC levels may not be informative due to the large number of non-disease-causing polymorphisms as well as high rates of VUS and pseudodeficiency variants [73].

To reduce the rate of false positives, a LC-MS/MS 2-TT has been developed to analyse psychosine concentrations on positive DBS samples. Several authors have reported higher levels of this biomarker in the DBS samples of infantile KD patients identified through NBS than in samples of newborns with normal GALC activity [74,75,76].

The combination of 2-TTs and post-analytical tools can reduce the incidence of false positives. Ratios of GALC activity to the other lysosomal enzyme activities are informative because they can distinguish true positives from true negatives [49].

## 5. Post-Analytical Tools

Several marker ratios are useful in the interpretation of NBS results. In screening for Phenylketonuria, the phenylalanine/tyrosine ratio has a greater clinical sensitivity and specificity than phenylalanine concentration alone [77]. This is one of the most informative ratios since, in the presence of the phenylalanine hydroxylase deficiency, phenylalanine is accumulated and tyrosine, the downstream product of the block, is decreased.

The C3/C2 ratio is another powerful indicator able to enhance the predictive performance of the MMA screening test. This ratio is usually used as a secondary parameter with a greater prognostic value than C3-carnitine levels alone. However, cutoff setting remain problematic. Low cutoff levels can result in large numbers of false positives while high cutoffs levels may lead to affected newborns being missed. Indeed, several affected newborns have been missed because both C3 and the C3/C2 ratio were within established normal ranges. The best approach is to lower cutoff levels so as not to miss affected newborns, while using 2-TT to reduce the number of false positives.

C8/C10 and C8/C2 ratios together with C8-carnitine levels are commonly used for predicting an MCAD diagnosis based on NBS results. The use of these ratios can aid the interpretation of milder alterations in acylcarnitines with medium chain. The acylcarnitine profile of severe MCAD deficiency is unequivocally recognizable but mild forms are less easy to identify.

Advances in the field of NBS have spurred the development of data collection platforms for the interpretation of metabolic profiles. Many screening laboratories used Region 4 Stork (R4S), active between 2004–2013 to interpret screening results.

R4S was developed to improve how screening results are interpreted by integrating abnormal values with additional clinical data. Analyte ranges for each pathological condition were established which were more informative than the single cutoff values used by individual laboratories [78]. The post-analytical tools provided by R4S allowed disease ranges used for NBS to be constantly updated. A score-condition was calculated using specific disease intervals for all informative analytes, leading to a reduction in the false positive rate and improved screening performance [78,79].

The advanced version of R4S, Collaborative Laboratory Integrated Reports (CLIR) extends the previous multivariate pattern-recognition software, by incorporating additional demographic information such as age, birth weight and gender which can be responsible for significant discrepancies among results collected by different labs [80].

CLIR collects large numbers of normal and positive screening profiles from NBS programs worldwide. The platform provides post analytical tools not only for the expanded NBS of metabolic disorders but also for other NBS programs. The availability, on a single platform, of data for all types of disease-related analytes including secondary biomarkers and analyte ratios as well as data on clinical variables has led to significant improvements in false-positive rates [79].

## 6. Molecular Testing as a 2-TT

The extraction of DNA from DBS allows for molecular testing in NBS programs [81]. Molecular analysis can confirm diagnoses and reduce the number of false positives.

Second-tier molecular testing was first introduced for conditions such as cystic fibrosis and sickle cell disease [82,83]. Screening for cystic fibrosis is performed through immunoreactive trypsinogen (IRT) analysis; this test has a high false positive rate, so most NBS cystic fibrosis laboratories perform second-tier genetic testing on specimens with elevated IRT levels [84]. Most laboratories use commercial kits that screen for a predefined mutation panel covering the more prevalent pathogenic variants. In recent years, many pilot projects have introduced DNA analysis into NBS before the family is alerted [85,86,87,88]. A retrospective study conducted in Pennsylvania evaluated the feasibility of a DNA-based 2-TT, covering the most common *GALT* mutations (seven mutations and two variants), to improve the specificity of NBS for Galactosemia. The approach seems useful for newborns with initial positive screening results, but it needs to be proven in prospective studies [85]. In the Netherlands, an NBS pilot program for X-Adrenonoleukodystrophy (X-ALD) has been drawn to screen only male newborns (X-ALD is more severe in male than female infants). DBS samples with elevated levels of C26:LPC (biomarker for X-ALD) in FIA-MS/MS and subsequently confirmed with separation by LC-MS/MS, undergo molecular analysis of all 10 exons of the gene. The project is designed to complete the NBS process within 5 weeks [88].

However, although this strategy can be used for a wide range of conditions, there remains some doubt over its application in disorders associated with acute metabolic decompensation. The potential benefit of a second-tier genetic test must be balanced against the potential harm in delaying recall for time-critical conditions.

Next-generation sequencing (NGS) technology allows sequencing of the whole genome, the whole exome or of a targeted gene panel to be performed. For inherited disorders, the first line of testing is usually a targeted gene panel that focuses on a specific cohort of genes. The feasibility of NGS as a second-tier diagnostic test in NBS has been studied [89].

Second-tier NGS testing using DNA from the same DBS after primary screening of TRECs was proposed also for severe combined immunodeficiency and a targeted next generation approach was considered as an available second-tier approach for Pompe disease when enzymatic activity on DBS is below the established cutoff values [48,86]. A feasibility study to improve NBS for cystic fibrosis using NGS has been also reported [90].

However, next-generation DNA sequencing technology raises several ethical considerations because of the detection of variants of uncertain clinical significance, the identification of carrier status and pseudodeficit or benign conditions without clinical manifestations [91]. Interpretation of genetic newborn screening results requires extensive knowledge of normal and pathogenic variants for every gene tested.

Other limits of these technologies are the high costs, the prolonged turnaround time and the identification of misclassified variants.

## 7. Conclusions

NBS has changed the natural history of several diseases and improves the quality of life for many patients. Effective NBS programs can bring significant cost-benefit advantages for public healthcare systems, but a high incidence of false positive results can cause serious and lasting psychological consequences for newborns and their families. It is, therefore, essential to develop more specific assays to investigate altered biomarker levels in primary screening results and to eliminate as many false positives as possible. The application of two or more strategies to improve the consistency of outcomes in NBS programs involves costs that can be well sustained by public health systems. Unfortunately, few NBS tests give a PPV near 100% but technological advances in methods are leading to important improvements. The aim of healthcare providers working in NBS systems should be to improve the positive predictive values of screening tests by applying appropriate analytical and post-analytical tools.

NBS programs should contain costs, prevent parental anxiety arising from results which are inconclusive or of unknown clinical significance and reduce the time needed to make a diagnosis.
